# COVID-19 Pandemic and Challenges Faced by Healthcare Professionals in India

**DOI:** 10.7759/cureus.38278

**Published:** 2023-04-29

**Authors:** Shibajee Debbarma

**Affiliations:** 1 Department of Community and Family Medicine, All India Institute of Medical Sciences, Patna, IND

**Keywords:** public sector, global healthcare systems, training of doctors, healthcare policy, mental health, personal protective equipment, violence, healthcare workforce, lockdown, health insurance

## Abstract

Since the Great Influenza Pandemic of 1918, a pandemic of such magnitude as the COVID-19 pandemic was yet to be confronted. While the pandemic led to unforeseen challenges globally as well as at the country level, it also brought forth certain perennial issues. This editorial is an attempt to revisit some of the major challenges faced by healthcare professionals in India during the pandemic. Timely interventions by the government of India dealt with several challenges confronted by the healthcare sector. However, issues about working hours, mental health, safety, and security of healthcare professionals also need to be looked into in the future.

## Editorial

Throughout history, the human race has been plagued by several infectious disease outbreaks. Since the Great Influenza Pandemic of 1918, which infected over 500 million people globally and led to more than 50 million deaths, a pandemic of such magnitude as the COVID-19 pandemic was yet to be confronted [1]. Following the outbreak of respiratory illness due to a novel coronavirus in China and with a rising number of cases across the world in early 2020, the government of India issued guidelines for screening and line listing of travelers from affected countries and for disease surveillance in states [2]. On 30^th ^January, World Health Organization (WHO) declared the outbreak a "public health emergency of international concern" [3]. It was the same day the first case of COVID-19 was reported in India, and two more cases followed soon after. The country's tally of three cases remained so for almost a month. This was a crucial period for the government to take stock of existing resources in the country's healthcare system and to take mitigatory measures.

The healthcare system in India is broadly divided into public and private sectors. Services in the public sector, that is, the government sector, are provided through a three-tier system comprising primary, secondary, and tertiary levels for ensuring healthcare coverage in rural as well as urban areas. The tertiary level contains teaching hospitals and advanced medical research institutions that observe huge footfall of patients daily. Resident doctors, namely, post-graduate trainees, senior residents, and junior residents, constitute a large proportion of healthcare service providers working in these tertiary-level institutions. A representative body, known as Resident Doctors' Association (RDA), exists in most of these institutions for highlighting and addressing their grievances. As a resident doctor as well as an office-bearer of a national federation of RDAs in 2020, along with firsthand experience of the pandemic, the author had the opportunity of working with colleagues from various states in dealing with emerging issues. It is worth mentioning that lessons from the past hold the key to success in the future. This editorial is, therefore, an attempt to revisit some of the major challenges faced by healthcare professionals, including resident doctors, in India during the first wave of the pandemic and the measures taken to deal with them, which may be adopted at the country level as well as globally, for future preparedness.

Safety of healthcare professionals and health insurance

In March 2020, COVID-19 cases were on the rise in India. The Union Health Ministry convened a meeting in mid-March with various associations of doctors, including representatives of the federation, regarding the situation. Noting the concerning reports of morbidities and mortalities among healthcare workers globally, the need to ensure the health and safety of healthcare professionals in the country was stressed during the meeting. Considering the risk involved at the workplace, the provision of an allowance, as per the existing risk and hardship allowance of Central Pay Commission (CPC) recommendations in India for high-risk jobs, was also discussed for ensuring the financial security of doctors in the public sector. It was highlighted that healthcare institutions should take up the responsibility of admission and treatment of their staff infected at the frontline. Necessary instructions in this regard were issued by the ministry for healthcare institutions [4]. An Insurance Scheme for Health Workers Fighting COVID-19 was subsequently announced by the government of India [5]. The scheme covered death among healthcare workers due to COVID-19 in addition to accidental death due to COVID-19-related duties.

Impact of lockdown and travel restrictions

A lockdown was imposed in India from 25^th^ March 2020, initially for a period of 21 days that was extended later on, to contain the spread of COVID-19 by breaking the chain of transmission [6]. Movement of people was prohibited across the country, except for those involved in essential services, and the travel pass was made compulsory for inter-state travel. Initially, availing of the pass was a cumbersome process, even for essential service providers. The delivery of services was impacted in several healthcare institutions, especially those located at inter-state borders. The problem was more severe in the national capital, Delhi, sharing its borders with two other states and with many of its healthcare staff residing in neighboring states. Shortage of healthcare workforce leading to prolonged duty hours and exhausted staff were being reported from several hospitals. Central and state authorities were approached for the solution to this acute crisis. Eventually, the Union Home Ministry instructed state authorities to ensure and facilitate the movement of healthcare professionals so that healthcare services remain unhindered across the country [7]. Though a short-lived issue, it highlighted the importance of having a streamlined system in place for the maintenance of essential services, especially during an emergency.

Management of the healthcare workforce

Being the second most populous country in 2020, it was necessary to tactfully manage the healthcare workforce of India to deal with the upcoming wave of the pandemic. Prior to the countrywide lockdown, the Union Health Ministry published guidelines for healthcare institutions for the management of existing resources and patient care [4]. Following the lockdown, the ministry, for the first time, published guidelines for the practice of telemedicine by registered medical practitioners in the country so that patients can benefit from online consultations and avoid visiting hospitals [8]. With the pandemic causing a rampage globally and with healthcare workers getting infected at the frontline, healthcare systems in several affected countries were already overwhelmed. Observing the global scenario, a plan of action was put forward by the federation to the Union Health Ministry that included suggestions for posting teams of doctors on a rotation basis for fixed duty hours in hospitals followed by a quarantine period so that adequate rest is ensured and chances of spread of infection are minimized. A standard operating procedure (SOP) for hospitals managing COVID-19 cases was subsequently issued by the ministry to deploy teams of healthcare staff in demarcated COVID-19, non-COVID-19, screening, and critical areas [9]. At the same time, special drives to recruit contractual healthcare staff, including doctors, were conducted by the authorities to support the existing ones. Though the healthcare system was stretched at the peak of the pandemic in the country, these measures were effective in managing the healthcare workforce as well as services during the course of the pandemic.

Ostracisation and violence against healthcare professionals

With an ever-rising number of COVID-19 cases globally and with people confined in their homes following the lockdown in India, a sense of fear and anxiety of contracting the infection gripped the country's population [10]. Initially hailed as "COVID warriors", soon there were reports of stigmatization of healthcare professionals in community settings. Healthcare professionals were being ostracised from several residential areas. On several occasions, colony gates were shut, and borders of villages closed for visiting healthcare teams, and in some areas, teams were even attacked by mobs. Though violence against doctors in India was on the rise prior to the pandemic, people's anxiety and frustration amidst the prevailing situation led to more frequent incidents. To curb this menace, the long-pending demand for a central act for the protection of doctors and other healthcare professionals was put forward by the federation to the government of India. Media personnel, political leaders, and influential persons were approached to create awareness among the masses. Looking at the alarming trend, the Union Home Ministry soon instructed state authorities to provide police security to healthcare professionals at the workplace and during community visits [11]. Authorities provided accommodations for doctors and other healthcare workers on COVID-19 duty, and advisories were issued for the population. Eventually, the Epidemic Diseases Act of 1897 was amended by the government to include the provision of strict punishment of offenders for acts of violence against healthcare personnel or damage to the property of healthcare institutions [12]. A commendable step during the pandemic, multiple incidents of violence, however, demand the implementation of provisions of the act beyond the pandemic in order to ensure the security of healthcare professionals even in the remotest corners of the country.

Supply of personal protective equipment

Being the largest manufacturer and exporter of personal protective equipment (PPE) kits in 2020, lockdown and other restrictions in China, in addition to the country's huge internal demand, led to an unprecedented shortage of kits globally. On the other hand, India was primarily an importer of PPE kits during that period. Anticipating the upcoming wave of the pandemic, the government of India was already working on the domestic manufacture of kits, which was in line with the Make in India initiative to ensure self-sustainability. The necessity of ensuring an adequate supply of quality PPE kits for frontline healthcare workers was also raised by several professional bodies. However, the country's production was not adequate to meet the huge demand during the initial days. Various organizations and societies came forward and provided PPE kits and other equipment to healthcare institutions across the country as a contribution to the national cause. With adequate government impetus and concerted efforts of all stakeholders, several domestic manufacturers and start-ups eventually came up, leading to an uninterrupted supply of PPE kits and other medical equipment within the country. From a mere importer, India soon became the second-largest producer of PPE kits globally, which was a massive boost for the domestic textile industry [13].

Emerging mental health issues

Globally, mental health issues, one of the leading causes of disability, have been on the rise much before the onset of the COVID-19 pandemic. The trend was no different in India, where the situation is complex due to a lack of awareness and stigma associated with mental health issues. Even under normal circumstances, doctors and other healthcare professionals are at increased risk of mental health issues due to the stressful nature of work. The situation was compounded by the pandemic with a high stress level at the workplace leading to depression, anxiety, and panic attacks [14]. Even some incidents of suicides were reported. Observing this epidemic of mental health issues amidst the ongoing pandemic, the federation collaborated with a professional body of psychiatrists, psychologists, and counselors who had set up a free pan-India teleconsultation service. Healthcare professionals across the country were able to consult mental health experts telephonically without disclosing their identity. Free online meditation sessions for healthcare professionals were also organized for stress management during those uncertain times. Eventually, a framework for mental health support of healthcare professionals published by a national mental health institution was adopted by the government of India for implementation across the country [15]. Special mental health clinics and helplines for the healthcare workforce were set up by several institutions. The pandemic, thus, brought into light an apparently hidden burden of mental health issues among healthcare professionals and the need to prioritize interventions along with risk communication.

Training of doctors

To tackle the pandemic, the government of India had advised healthcare institutions to postpone non-emergency services till the situation normalizes [4]. Patients were also advised to utilize telemedicine services in order to reduce footfall in hospitals. Healthcare resources were diverted toward the management of COVID-19 cases, which was the need of the situation. However, with the first wave of the pandemic prevailing for months and with the deferment of elective procedures and other services, the necessary training of resident doctors and research work was impacted. As the situation gradually normalized, the National Medical Commission was approached for relaxation in medical education and training guidelines and for allowing resident doctors to continue their training even after completion of tenure to acquire the necessary skills. Considering the extraordinary circumstances, the commission modified several guidelines related to research work, dissertation, conduction of examination, etc., as a one-time measure. Also, considering the impact on training and ensuring the availability of an adequate healthcare workforce, willing resident doctors were allowed by authorities to work in respective institutions at existing salaries following tenure completion.

Conclusion

While the COVID-19 pandemic led to unforeseen challenges globally as well as at the country level, it also brought forth certain perennial issues faced by healthcare professionals. The pandemic highlighted the importance of having an adequate and healthy workforce in place for delivering quality healthcare services. Timely interventions by the government of India effectively dealt with several challenges confronted by the healthcare sector during the pandemic. However, issues pertaining to working hours, mental health, safety and security of healthcare professionals need to be looked into, in the future as well. The necessity of creating a separate service cadre for doctors, the Indian Medical Service cadre, has been highlighted previously by several health committees, experts, and associations of doctors so that the country's healthcare sector is managed by subject experts having adequate theoretical and practical knowledge [16]. Though the need for the expertise of doctors in devising healthcare policies and strategies was felt even more during the COVID-19 pandemic, such a cadre is yet to see the light of day. Along with an increment in resource allocation, lessons from the pandemic need to be incorporated into the national health policy for the overall improvement of the country's healthcare sector.
